# Clinical characteristics and outcomes of B-cell precursor ALL with *MEF2D* rearrangements: a retrospective study by the Ponte di Legno Childhood ALL Working Group

**DOI:** 10.1038/s41375-022-01737-4

**Published:** 2022-10-29

**Authors:** Kentaro Ohki, Ellie R. Butler, Nobutaka Kiyokawa, Shinsuke Hirabayashi, Anke K. Bergmann, Anja Möricke, Judith M. Boer, Hélène Cavé, Giovanni Cazzaniga, Allen Eng Juh Yeoh, Masashi Sanada, Toshihiko Imamura, Hiroto Inaba, Charles G. Mullighan, Mignon L. Loh, Ulrika Norén-Nyström, Lee-Yung Shih, Marketa Zaliova, Ching-Hon Pui, Oskar A. Haas, Christine J. Harrison, Anthony V. Moorman, Atsushi Manabe

**Affiliations:** 1 Department of Pediatric Hematology and Oncology Research, National Research Institute for Child Health and Development, Tokyo, Japan; 2 Leukaemia Research Cytogenetics Group, Wolfson Childhood Cancer Research Centre, Translational and Clinical Research Institute, Newcastle University, Newcastle upon Tyne, UK; 3 Department of Pediatrics, Hokkaido University Graduate School of Medicine, Sapporo, Japan; 4 Hannover Medical School, Institute of Human Genetics, Hannover, Germany; 5 Department of Pediatrics, Christian-Albrechts-University Kiel and University Medical Center Schleswig-Holstein, Kiel, Germany; 6 Princess Máxima Center for Pediatric Oncology, Utrecht, The Netherlands; 7 Oncode Institute, Utrecht, The Netherlands; 8 Department of Genetics, Robert Debré Hospital and Université Paris Cité, Paris, France; 9 Centro Ricerca Tettamanti, Pediatrics, University of Milano Bicocca, Monza, Italy; 10 Medical Genetics, School of Medicine and Surgery, University of Milano Bicocca, Monza, Italy; 11 Khoo Teck Puat - National University Children’s Medical Institute, Yong Loo Lin School of Medicine, National University of Singapore, Singapore, Singapore; 12 Department of Advanced Diagnosis, Clinical Research Center, National Hospital Organization Nagoya Medical Center, Nagoya, Japan; 13 Department of Pediatrics, Graduate School of Medical Science, Kyoto Prefectural University of Medicine, Kyoto, Japan; 14 Department of Oncology, St Jude Children’s Research Hospital, Memphis, TN USA; 15 Department of Pathology, St Jude Children’s Research Hospital, Memphis, TN USA; 16 Department of Pediatrics, Benioff Children’s Hospital and the Helen Diller Family Comprehensive Cancer Center, University of California, San Francisco, San Francisco, CA USA; 17 Department of Clinical Sciences, Pediatrics, Umeå University, Umeå, Sweden; 18 Division of Hematology-Oncology, Chang Gung Memorial Hospital at Linkou and Chang Gung University, Taoyuan, Taiwan; 19 CLIP, Department of Paediatric Haematology/Oncology, Second Faculty of Medicine of Charles University Prague and University Hospital Motol, Prague, Czech Republic; 20 Children’s Cancer Research Institute, Vienna, Austria

**Keywords:** Acute lymphocytic leukaemia, Oncogenesis

## To the Editor:

B-cell precursor acute lymphoblastic leukemia (BCP-ALL) comprises multiple genetic subtypes with strong prognostic associations. The outcome of patients with high-risk genetics improves with risk stratification or targeted therapy [1, 2]. Therefore, it is important to assess the prognostic impact of newly identified genetic abnormalities to ensure appropriate clinical intervention. Recent studies have identified *MEF2D* rearrangements (*MEF2D-*r*)* in 2–3% cases and initial observations, based on small numbers of cases, indicate that patients have a poor outcome [3–7]. *MEF2D*-r are characterized by fusion of an N-terminal region of *MEF2D* to the C-terminal region of multiple, different partner genes [3–8]. As there are limited data available concerning the prognostic impact of *MEF2D*-r in ALL, we conducted an international study via the Ponto di Legno Childhood Leukemia Working Group to describe the clinical characteristics and outcome of patients with BCP-ALL and *MEF2D*-r.

Demographic, clinical, treatment, genetics and outcome data were collected from 14 regional study groups (Supplementary Table 1). Patients were diagnosed between 1987 and 2018 and *MEF2D*-r were detected retrospectively, using a range of techniques (Supplementary Table 2). The majority of cases [97/107(91%)] were identified by screening diagnostic samples from representative cohorts of B-other-ALL (i.e. patients lacking an established genetic abnormality). Additional cases were identified among relapse patients and/or in relapse samples. These cases were excluded from the survival analysis (*n* = 10). We considered three endpoints: relapse rate (RR), event-free survival (EFS) and overall survival (OS), using the Kaplan-Meier method, log-rank test and Cox regression models, retrospectively, as previously described [9]. All rates are quoted at 5 years.

Among 107 *MEF2D*-r patients, there was female predominance (66:41) with a median age of 10.67 years (Table 1). A quarter of patients had diagnostic peripheral blood white blood cell (WBC) counts >50,000/μl, which, coupled with the older age, resulted in 70% (60/98) being classified as National Cancer Institute (NCI) high risk. Data on antigen expression were available for 91 of the 107 cases. Different panels were used so the amount of data was variable for each antigen (Supplementary Table 3). Cases distributed evenly across EGIL groups: pro-B, pre-B, late pre-B. HLA-DR, cytoplasmic immunoglobulin μ chain, CD45, CD22 and CD19 were commonly expressed (>80% tested cases). CD10 and CD5 were expressed in 65% and 56% tested cases, respectively. None of the other tested antigens (CD2, CD3, CD7, CD13, CD20, CD33, CD34 and CD66c) were expressed in >20% tested cases. Unfortunately, data were unavailable for CD38 expression, which has reported to be a feature of *MEF2D*-r [3–8]. Although we did not include a comparator cohort, we confirmed the distinct features associated with *MEF2D*-r reported in smaller studies [3–8]. Namely female sex, older age, common expression of cytoplasmic immunoglobulin μ chain and CD5, and less frequent expression of CD10.

**Table 1:** Demographic and outcome features of patients with BCP-ALL and a MEF2D rearrangement stratified by partner gene.

	Total	*BCL9*	*HNRNPUL1*	*p* value^a^ *BCL9 v HNRNPUL1*	Other^b^	*p* value^a^ *BCL9* v *HNRNPUL1* v other	Missing
Total, *n* (%)	107 (100)	37 (54)	22 (32)	–	10 (14)		38
Sex, *n* (%)							
Male	41 (38)	13 (35)	11 (50)	0.3	2 (20)	0.2	15 (39)
Female	66 (62)	24 (65)	11 (50)		8 (80)		23 (61)
Age at initial diagnosis(years)							
Median	10.67	9.48	9.09		8.91		12.00
1–9	40 (42)	16 (53)	11 (52)	0.2	5 (50)	0.2	8 (23)
10–14	41 (43)	8 (27)	9 (43)		5 (50)		19 (54)
15–18	15 (16)	6 (20)	1 (5)		0 (0)		8 (23)
Unknown/Missing	11	7	1		0		3
WBC Count (10^6^/L) at diagnosis							
<50,000	78 (74)	26 (72)	20 (91)	0.09	5 (50)	0.04	27 (71)
>50,000	28 (26)	10 (28)	2 (9)		5 (50)		11 (29)
Unknown/Missing	1	1	0		0		0
NCI risk group at diagnosis							
Standard risk	29 (30)	11 (35)	10 (48)	0.4	3 (30)	0.6	5 (14)
High risk	69 (70)	20 (65)	11 (52)		7 (70)		31 (86)
Missing	9	6	1		0		2
CNS disease at diagnosis (CNS3)							
Yes	4 (4)	0 (0)	1 (6)	0.4	1 (11)	0.3	2 (6)
No	85 (96)	27 (100)	17 (94)		8 (89)		33 (94)
Unknown/Missing	18	10	4		1		3
Year of Diagnosis							
1992–2007	52 (49)	15 (41)	9 (41)	0.9	6 (60)	0.5	22 (58)
2008–2018	55 (51)	22 (59)	13 (59)		4 (40)		16 (42)
Race							
Asian	29 (45)	14 (61)	12 (75)	0.4	3 (60)	0.6	0 (0)
White	28 (43)	7 (30)	2 (13)		2 (40)		17 (81)
Other	8 (12)	2 (9)	2 (13)		0 (0)		4 (19)
Unknown/Missing	42	14	6		5		17
Treatment risk groups							
Non-high risk	60 (56)	23 (62)	15 (68)	0.4	8 (80)	0.6	14
High risk	47 (44)	14 (38)	7 (32)		2 (20)		24
Minimal residual disease at end of induction							
Positive (≥0.01%)	2 (7)	1 (9)	0 (0)	0.4	1 (25)	0.4	0 (0)
Negative (<0.01%)	26 (93)	10 (91)	6 (100)		3 (75)		7 (100)
Unknown/Missing	79	26	16		6		31
Stem cell transplant Received							
Yes	2 (3)	2 (6)	0 (0)	0.2	0 (0)	0.4	0 (0)
No	73 (97)	31 (4)	22 (100)		8 (100)		38 (100)
Unknown/missing	32	3	0		2		26
Outcome analysis							
Cases included^c^	95 (100)	33 (55)	18 (30)	–	9 (15)		35
Median followup (years)	6.73	6.16	6.75	0.7	5.79	0.6/0.7^d^	6.97
5 years survival, % (95% CI)							
Relapse rate	24% (16–35)	32% (18–53)	6% (1–37)	0.07	13% (2–61)	0.2	29% (16–48)
Event-free	74% (63–82)	65% (45–80)	94% (63–99)	0.05	88% (39–98)	0.1	68% (49–82)
Overall	81% (71–88)	76% (55–88)	94% (63–99)	0.2	88% (39–98)	0.3	78% (59–89)
Site of relapse							
Bone marrow (BM)	13 (68)	7 (78)	1 (100)	0.9	1 (50)	0.9	4
BM + CNS	2 (11)	1 (11)	0 (0)		1 (50)		1
CNS	3 (16)	1 (11)	0 (0)		0 (0)		2
Other	1 (5)	0 (0)	0 (0)		0 (0)		1
Missing	2	0	0		1		1
Univariate Cox Model (hazard ratio (95% confidence interval), *p* value)							
Relapse	–	1	0.18 (0.02–1.43)	0.1	–		–
Event	–	1	0.16 (0.02–1.28)	0.09	–		–
Death	–	1	0.24 (0.03–1.97)	0.2	–		–

^a^*P* values are from Chi-squared test, t-test, log rank test or Cox regression model as appropriate.

^b^The other group includes 4 cases of *FOXJ2* and 1 case each of *BCL9L*, *HNRNPH1*, *PYGO2*, *SS18*; plus two cases of *CSFR1*.

^c^Twelve patients were excluded because they had missing data (*n* = 2), had been selected for screening because they had relapsed or had refractory disease (*n* = 7) or the fusion had only be detected at relapse (*n* = 3).

^d^*BCL9* v Other and *HNRNPUL1* v Other.

Information on the fusion partner gene was available for 69/107 (64%) patients. *MEF2D::BCL9* and *MEF2D::HNRNPUL1* were the most common fusions, detected in 37 and 22 cases, respectively; together accounting for 85% cases. Other partner genes were identified as follows: *FOXJ2* (*n* = 4, 6%), *CSF1R* (*n* = 2, 3%), and single cases of *HNRNPH1*, *PYGO2, BCL9L*, and *SS18*. The partner gene was not determined in the remaining 38 cases due to lack of suitable material or unavailability of a relevant technique. There were no statistical differences in the distribution of age, NCI risk group, ethnicity, or leukocyte count according to partner gene (Table 1). Due to lack of data, we are unable to confirm a recent observation, showing that black patients had a higher incidence of *MEF2D*-r [10]. The distinctive antigen profile associated with *MEF2D*-r (cytoplasmic immunoglobulin μ chain and CD5) was consistent in cases with different partner genes. However, patients with *MEF2D::HNRNPUL1* were significantly more likely to express CD10 compared to patients with *MEF2D::BCL9* [18/20(90%) v 17/32(53%), *p* = 0.007) and be late pre-B [11/18(61%) v 8/31(26%), *p* = 0.016] (Supplementary Table 3).

Approximately 60% cases were tested by MLPA/SNP arrays for deletions affecting *IKZF1*, *PAX5*, *CDKN2A/B*, and *ETV6*, whilst a smaller number were screened for mutations in *NRAS/KRAS*, *FLT3*, *NOTCH1*, *FBXW7* and *PHF6* (Supplementary Table 4). The most common secondary abnormality was *CDKN2A/B* deletion, occurring in 48/68 (71%) cases. *PAX5*, also located to 9p, was co-deleted in 12 cases. *IKZF1* deletions and *NRAS/KRAS* mutations were rare, occurring in ≤10% cases. As previously noted, the frequency of *PHF6* mutations, usually associated with T-ALL, was high (25%) [7]. There was little evidence that the frequency of secondary copy number alterations or mutations varied in relation to partner gene. However, 0/18 cases with *MEF2D::HNRNPUL1* carried a *PAX5* deletion. The spectrum of secondary alterations in *MEF2D*-r patients was not typical of B-other-ALL. The proportion of patients with *CDKN2A/B* deletions was much higher than expected, whilst the frequency of *IKZF1* deletions was lower [11].

**Fig. 1:**
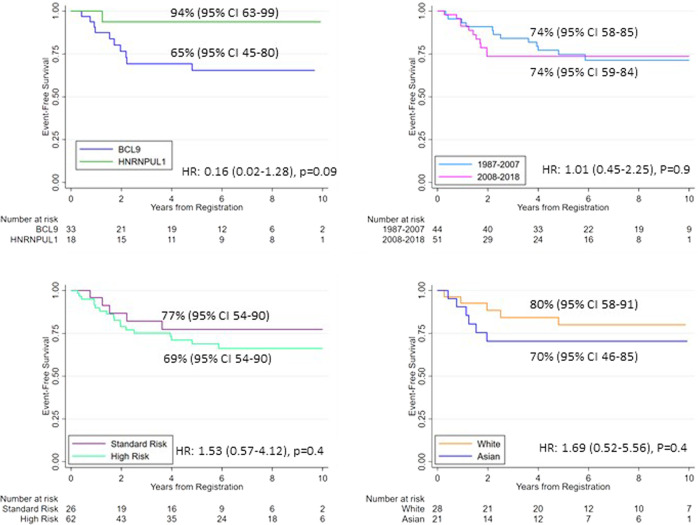
Event free survival of patients with B-cell precursor ALL and *MEF2D* rearrangements stratified by partner gene, period of diagnosis, NCI risk group and ethnicity. All event free survival rates are quoted at 5 years with accompanying 95% Confidence Intervals. HR hazard ratio, NCI National Cancer Institute.

Outcome data for 95 patients with *MEF2D*-r was available for analysis (Table 1). All patients achieved a complete hematological remission and 26/28 (93%) cases tested were MRD negative (<0.01%) at the end of remission induction therapy. Despite this good early response, 39/95 (44%) cases were treated on the high-risk protocols of each study group, likely reflecting the observation that most patients were NCI high-risk. Very few patients (2 of 75, 3%) received a hematopoietic stem cell transplant, consistent with 90% cases being MRD negative at the end of induction. After a median follow-up time of 6.73 years, the EFS rate was 74% (63%–82%) with corresponding relapse and survival rates (Table 1). The majority of relapses (79%) involved bone marrow. There was no significant difference in EFS by NCI risk status, treatment period (pre- and post- 2008) or race (white vs Asian) (Fig. 1, Supplementary Table 5). Our cohort was incomplete for data on race in terms of both classification and numbers of cases, so only very large differences in outcome would be detectable.

Patients with *MEF2D::HRNPUL1* had an EFS of 94% (95% CI 63–99), which was numerically, but not statistically significantly, higher than the EFS for patients with *MEF2D::BCL9* − 65% (95% 45–80) (log rank test *p* = 0.05). A univariate Cox model comparing the risk of an event among *MEF2D::HRNPUL1* cases with *MEF2D::BCL9* cases revealed a hazard ratio of 0.16 (95% CI 0.02–1.28), *p* = 0.09 (Table 1). The trend towards a better outcome for patients with *MEF2D::HRNPUL1* correlated with the high frequency CD10 expression and proportion of late pre-B cases in this subtype. Both factors were also linked with better outcome: CD10 expression (yes v no) EFS 83% (95% 64–93) v 57% (27–78), log-rank *p* = 0.04; late pre-B v pro-B/pre-B 94% (63–99) v 63% (41–78) log-rank *p* = 0.03. Nine of 33 patients with *MEF2D::BCL9* relapsed with a median time to relapse of 20 months. Only 1/18 patients with *MEF2D::HRNPUL1* relapsed. CD5 expression was highest among patients with *MEF2D::BCL9* (64%) but there was no difference in outcome between patients expressing and not expressing CD5: EFS 64% (95% 36–82) v 79% (47–93), log rank test *p* = 0.3. Our ability to examine the prognostic effect of secondary abnormalities was limited both by the number of cases tested, as well as the rarity of recurrent abnormalities. However, there was no significant prognostic effect of the presence of either *CDKN2A/B* or *PAX5* deletions (log rank *p* values >0.2 for all three endpoints).

Previous studies, based on small numbers of cases, have reported the therapeutic outcome of *MEF2D*-r BCP-ALL to be unfavorable. For example, analysis of NCI-high risk children enrolled on AALL0232 showed that 20 *MEF2D*-r cases belonged to the group with EFS of 72%, which was comparable to *BCR::ABL1* (60%), *KMT2A-r* (78%) and Ph-like (60%), but lower than other BCP-ALL cases (87%) [6]. In the TCCSG L04‑16 Study, the EFS and OS rates for BCP-ALL patients was 80% and 92%, respectively, but 50% and 56% respectively for *MEF2D*-r cases [7]. The major strength of this study is that it collected a large, well-annotated cohort of *MEF2D*-r cases. Although the patients were not uniformly treated, they did not exhibit significant outcome heterogeneity by era or NCI risk status. In this study, 24% patients had relapsed and the EFS was 74%, indicating the therapeutic outcome of *MEF2D*-r, whilst not extremely poor, was lower than expected for patients with intermediate risk genetics. In this study, patients with *MEF2D::BCL9* had an EFS of 65%, close to the rates reported in AALL0232 and TCCSG L04-16 studies that were predominantly based on *MEF2D::BCL9* cases. In contrast, only 6% (*n* = 1) of *MEF2D::HNRNPUL1* cases in this study had relapsed within 5 years and the EFS was 94%. There was no difference in the distribution or outcome of *MEF2D::HNRNPUL1* or *MEF2D::BCL9* patients by NCI risk status. Although a direct comparison of the outcome of patients with *MEF2D::HNRNPUL1* or *MEF2D::BCL9* did not reach statistical significance, the large numerical differences in relapse and EFS do indicate outcome heterogeneity according to partner gene.

In conclusion, this retrospective multi-center study confirmed that *MEF2D* fusions are associated with female sex, older age and atypical immunophenotype. The most common fusion partners were *BCL9* and *HNRNPUL1*, accounting for >80% cases. We have confirmed previous studies that suggest a high risk of relapse for patients with *MEF2D::BCL9* fusions, but we could not confirm that this poor outcome extended to patients with other *MEF2D* partners.

## Supplementary information

Supplementary Tables

## Data Availability

The datasets generated during and/or analyzed during the current study are available from the Ponte di Legno Childhood ALL Working Group via the corresponding author on reasonable request.
